# Matrine suppresses cell growth of diffuse large B-cell lymphoma via inhibiting CaMKIIγ/c-Myc/CDK6 signaling pathway

**DOI:** 10.1186/s12906-021-03315-0

**Published:** 2021-06-04

**Authors:** Jianyou Gu, Xiao Wang, Ling Zhang, Jingjing Xiang, Jingya Li, Zheng Chen, Yu Zhang, Junfa Chen, Jianping Shen

**Affiliations:** 1 The First Affiliated Hospital, Zhejiang Chinese Medical University, No. 54 Youdian Road, Hangzhou, 310006 Zhejiang China; 2 Key Laboratory of Integrative Chinese and Western Medicine for the Diagnosis and Treatment of Circulatory Diseases of Zhejiang Province, No. 54 Youdian Road, Hangzhou, 310006 Zhejiang China

**Keywords:** Matrine, DLBCL, C-Myc, CaMKIIγ, CDK6

## Abstract

**Background:**

C-Myc aberrations confer a more aggressive clinic behavior in diffuse large B-cell lymphoma (DLBCL). Matrine is an alkaloid extracted from *Sophora flavescens* Ait. It possesses anti-cancer property through inhibiting the cell proliferation and inducing the apoptosis. The present study aimed to explore the underlying mechanisms of matrine in suppressing the cell growth of DLBCL.

**Methods:**

The influence of matrine on the viability of cultured DLBCL cell lines SU-DHL-16 and OCI-LY3 cells were determined by CCK-8. Apoptosis and cell cycle were measured by flow cytometry after matrine exposure. Western blot was taken to investigate the expression of activated Caspase-3, cleaved PARP, c-Myc, phospho-c-Myc (Ser62), CaMKIIγ, phospho-CaMKIIγ (Thr287), CDK4 and CDK6 after matrine treatment. Cycloheximide chase analysis was used to determine the c-Myc protein half-lives before and after matrine treatment. Growth salvage analysis was taken by ectopic expression of c-Myc.

**Results:**

In cultured DLBCL cells, matrine suppressed cell viability in a concentration and time dependent fashion. Matrine treated SU-DHL-16 and OCI-LY3 cells for 48 h with IC_50_ value of 1.76 mM and 4.1 mM, respectively. Matrine induced apoptosis through a caspase-independent pathway and caused G_0_/G_1_ cell cycle arrest in a concentration dependent manner in DLBCL cells. The protein expression of c-Myc was inhibited while the transcription of c-Myc was not reduced by matrine. c-Myc protein half-lives were decreased from 30.4, 69.4 min to 16.6, 15.9 min after matrine treatment in SU-DHL-16 and OCI-LY3, respectively. As a critical protein kinase of c-Myc, CaMKIIγ phosphorylation at Thr287 was found to be down-regulated and c-Myc phosphorylation at Ser62 was reduced together after matrine treatment in DLBCL. The growth suppression of SU-DHL-16 cells induced by matrine was rescued by over-expression of c-Myc achieved by recombinant adenovirus infection. The decreased expression of CDK6, not CDK4, induced by matrine was rescued by ectopic expression of c-Myc protein.

**Conclusions:**

This study has shown for the first time that matrine suppresses cell growth of DLBCL via inhibiting CaMKIIγ/c-Myc/CDK6 signaling pathway.

**Supplementary Information:**

The online version contains supplementary material available at 10.1186/s12906-021-03315-0.

## Background

Diffuse large B-cell lymphoma (DLBCL) is the most common class of non-Hodgkin lymphomas with a highly heterogeneous group of diseases [1]. According to cell-of-origin determined by immunohistochemical expression of CD10, BCL6, and IRF4/MUM1, DLBCL can be assorted into two subtypes: germinal center B-cell lymphoma subtype and active B-cell/non-germinal center B-cell lymphoma [2]. The gold standard treatment for DLBCL patients is R-CHOP regimen (rituximab plus cyclophosphamide, doxorubicin, vincristine, and prednisone) offering a 5-year overall survival of around 65%. Nonetheless, about 35% patients develop relapsed/refractory disease after initial response, with 70% of the patients dying from lymphoma within the first 2 years after disease progression [3]. Only 10% of refractory/relapsed DLBCL patients can be cured by high-dose therapy with autologous stem cell transplantation [4]. The remaining 90% of patients are incurable, which demands new targeted agents for clinical trials.

MYC is widely expressed in the normal cells and the expression levels tightly correlate to cell proliferation [5]. c-Myc functions as potent transcription factor, binding to the majority of regulated genes in the genome. Deregulation of the c-Myc is a typical feature of cancer initiation and maintenance [6]. MYC dysregulation is an important characterized oncogenic event in DLBCL. MYC overexpression is detected in about 40% of DLBCL [7]. MYC translocation is confirmed in 7 to 21% of DLBCL and these alternations are frequently associated with BCL2 or BCL6 rearrangements. MYC mutations exist in about 33.3% of DLBCL. Increased MYC gene copy number was detected in 8 to 20% of DLBCL. MYC aberrations in DLBCL confer a more aggressive clinical behavior with poor prognosis [8]. c-Myc protein is intrinsically disordered and has no globular functional domains [6]. It is a big challenge to directly inhibit the function of c-Myc.

As an important part of complementary medicine, Chinese herbal medicines have been used as adjuvant treatment for cancer in China and other countries [9]. Matrine is a naturally occurring alkaloid compound extracted from Chinese herbs like *Sophora flavescens*. It possesses anti-inflammatory, anti-oxidant, antiviral and anti-allergic properties [10]. Recently, several reports showed that matrine has anti-hematological malignancy activities against leukemia, multiple myeloma, NK/T-cell lymphoma (NKTCL) [11–14]. However, the effect of matrine on DLBCL is still unknown. Present study investigated the antitumor effect of matrine in human DLBCL cells and its related molecular mechanism.

## Methods

### Cell culture and reagents

Human DLBCL cell lines SU-DHL-16 (germinal center B-cell lymphoma subtype) and OCI-LY3 (active B-cell lymphoma subtype) were purchased from the DSMZ collection (Germany) and cultured in RPMI 1640 medium supplemented with 10 and 15% fetal bovine serum (Gibco, USA) at 37 °C with 5% CO_2_ atmosphere, respectively. Vindesine sulfate was ordered from Hangzhou Minsheng Pharmaceutical Co., Ltd. (China) and dissolved in 0.9% NaCl. Matrine was obtained from Nanjing Zelang Medical Technology Co., Ltd. (China) and dissolved in RPMI 1640 medium. Cell Counting Kit-8 (CCK-8) was purchased from Good Laboratory Practice Bioscience (USA). Dead Cell Apoptosis Kit (Cat# V13241) and TRIzol were ordered from Invitrogen (USA). Cell Cycle Analysis Kit was bought from Beyotime (China). Cycloheximide (CHX) was bought from Cayman Chemical (USA). HiScript II Q RT reagent kit and ChamQ™ SYBR qPCR Master Mix kit were bought from Vazyme (Nanjing, China). The antibody for c-Myc (Cat# ab32072) was ordered from Abcam (USA). The antibodies for phospho-c-Myc (Ser62) (Cat# 13748), PARP (Cat# 9532), Caspase-3 (Cat# 9662), CDK4 (Cat# 12790) and CDK6 (Cat# 13331) were purchased from Cell Signaling Technology (USA). Ca^2+^/calmodulin-dependent protein kinase II γ (CaMKIIγ) antibody (Cat# AP7208a) was ordered from Abgent (Suzhou, China). Phospho-CaMKIIγ (Thr287) antibody (Cat# abs131059) was purchased from Absin (Shanghai, China). GAPDH antibody (Cat# 60004–1-Ig) was bought from Proteintech (USA). Prestained and western blot marker was ordered from Haigene (Harbin, China). The recombinant adenovirus for c-Myc over-expression (HBAD-h-MYC-1 × flag-EGFP) and control (HABD-EGFP) were purchased from Hanbio Biotechnology (Shanghai) Co., LTD.

### Cell viability analysis

The cell viability was determined by CCK-8 assay. About 40,000 SU-DHL-16 and OCI-LY3 cells were plated into each well of 96 well plates, respectively. Cells were treated by increasing concentrations of matrine (125, 250, 500, 1000, 2000, 4000 μM for SU-DHL-16; 250, 500, 1000, 2000, 4000, 8000 μM for OCI-LY3) for 24 h, 48 h and 72 h, and vindesine sulfate (positive control) (0.00004, 0.0002, 0.001, 0.005, 0.025 μM) for 72 h. Negative control cells were treated with medium only. CCK-8 was added for the viability assay. The absorbance of the solution was read by a microplate reader Epoch 2 (BioTek, USA), using a test wavelength of 450 nm. Results obtained were expressed as percentage inhibition rate to test agents. GraphPad Prism program (USA) was used to calculate half maximal inhibitory concentration (IC_50_).

### Flow cytometric analysis for apoptosis

Approximately 5 × 10^5^ SU-DHL-16 and OCI-LY3 cells were seeded into each well of six well plates, respectively. Cells were incubated with increasing concentrations of matrine (1, 2, 4 mM for SU-DHL-16; 2, 4, 8 mM for OCI-LY3) for 48 h. Cells mixed with medium only were used for the control. The cells were washed with chilled PBS and then resuspended in 1 × binding buffer. Each sample was incubated with 5 μL Alexa Fluor 488 annexin V and 1 μL 100 μg/mL PI for 15 min in the dark at room temperature. The samples were analyzed within 1 hour by flow cytometry (Navios, Beckman Coulter, USA).

### Cell cycle analysis

SU-DHL-16 and OCI-LY3 cells (5 × 10^5^) were planted into six well plates, respectively. Increasing concentrations of matrine (0.5, 1, 2 mM for SU-DHL-16; 1, 2, 4 mM for OCI-LY3) were used to treat cells for 24 h. Cells with medium only were used for the control. The cells were collected and then fixed in 70% ice-cold ethanol at 4 °C for overnight. Cells were washed again by cold PBS and suspended in PBS containing 0.1 mg/mL RNase A and 50 μg/mL PI for 30 min at room temperature. DNA contents of cells were determined with flow cytometry within an hour. Cell cycles were resolved by ModFit LT Version 3.1 software (USA).

### Quantitative RT-PCR analysis

About 7.5 × 10^5^ SU-DHL-16 and OCI-LY3 cells were implanted into 6-well plates, respectively. Cells were incubated with different concentrations of matrine (1.2, 2.4, 3.6 mM for SU-DHL-16; 2, 4, 6 mM for OCI-LY3) for 24 h. Cells with medium only were taken as the control. The cells were collected. Total RNA extraction, cDNA synthesis and qPCR were performed as described previously [14]. Briefly, total RNA was isolated by TRIzol. cDNAs were synthesized from total RNA with the HiScript II Q RT reagent kit. The ChamQ™ SYBR qPCR Master Mix kit was applied for real-time reverse transcription PCR according to the manufacturer’s instructions in the CFX384 Real Time PCR system (Bio-Rad, USA). PCR conditions were: initial denaturation at 95 °C for 3 min followed by 40 cycles: 95 °C, 10s and 60 °C, 30s. After amplification, a melting-curve analysis was performed. The threshold cycle (Ct) was calculated using default threshold settings. GAPDH and β-Actin were used as the double-control to normalize mRNA input. The relative gene expression quantification was determined by the 2^−ΔΔct^ method. All experiments were done in triplicates. The primers used were as follows. c-Myc: 5′-CAGCTGCTTAGACGCTGGATT-3′ (forward) and 5′-GTAGAAATACGGCTGCACCGA-3′ (reverse); GAPDH: 5′-GGAGCGAGATCCCTCCAAAAT-3′ (forward) and 5′-GGCTGTTGTCATACTTCTCATGG-3′ (reverse); β-Actin: 5′-CACCTTCTACAATGAGCTGCGTGTG-3′ (forward) and 5′-ATAGCACAGCCTGGATAGCAACGTAC-3′ (reverse).

### Western blot

After matrine treatment for indicated time, DLBCL cells were washed with chilled PBS buffer. Total cellular proteins extraction, SDS-PAGE and membrane transfer were processed step by step. Western blot was taken as described previously [14]. Briefly, membranes with proteins were blocked with 5% nonfat milk in TBS–Tween 20 (TBST). The membranes were then incubated with primary antibodies overnight at 4 °C. After 3 washes with TBST, membranes were bound with a horseradish peroxidase-conjugated secondary antibody for 1 h at room temperature, and developed with Immobilon Western Chemiluminescent HRP Substrate (Millipore, USA). Protein levels were determined using Odyssey Fc Imager (LI-COR, USA) with the densitometric intensity by Image Studio software (USA).

### Cycloheximide chase analysis

Approximately 1.25 × 10^6^ SU-DHL-16 and OCI-LY3 cells were treated with or without 1.76 mM and 4.1 mM matrine for 12 h, respectively. 100 μg/mL cycloheximide was then added and cells were collected at indicated time points, and western blotting was done as above description.

### Growth salvage assay by ectopic expression of c-Myc

The recombinant adenovirus for c-Myc over-expression (HBAD-h-MYC-1 × flag-EGFP) and control (HABD-EGFP) were used for growth salvage analysis. The ectopic expression of EGFP and optimal multiplicity of infection (MOI) were confirmed by fluorescence microscope. OCI-LY3 failed and only SU-DHL-16 was infected successfully by recombinant adenovirus. The optimal MOI was 600. After that, about 40,000 SU-DHL-16 cells in 50 μL per well were plated into 96 well plates. Cells were divided into six groups as Control (RPMI 1640 media only), Control virus (MOI = 600), Myc virus (MOI = 600), Matrine (1.76 mM matrine), Matrine/Control virus (1.76 mM matrine with control virus at 600 MOI) and Matrine/Myc virus (1.76 mM matrine with Myc virus at 600 MOI). Blank group was set without cells and added with RPMI 1640 media. Each group had 6 replicates. Adenovirus or RPMI 1640 medium was added in 10 μL per well. The plates were horizontally centrifuged at 200×g for 1 h at room temperature. Plates were then incubated at 37 °C and 5% CO_2_ for 3 h. Matrine or RPMI 1640 medium was added in 40 μL per well. Cells were then incubated at 37 °C and 5% CO_2_ for 48 h. Cell survival was detected by CCK-8 dye at 48 h.

For western blot, 1 × 10^6^ SU-DHL-16 cells were implanted into six well plates. Cells were treated with matrine and adenovirus as above protocol. After 48 h, the cells were collected. The protein expressions of c-Myc, CDK4 and CDK6 were confirmed by western blot as above description.

### Statistical analysis

Data were expressed as mean ± standard deviation. Student’s t-test was applied for comparison of the means of two groups, and one way Analysis of Variance (ANOVA) was used to assess the level of significance between the means of multiple groups. Statistical significance was defined as *p* < 0.05.

## Results

### Matrine inhibits the growth of DLBCL cells

**Fig. 1:**
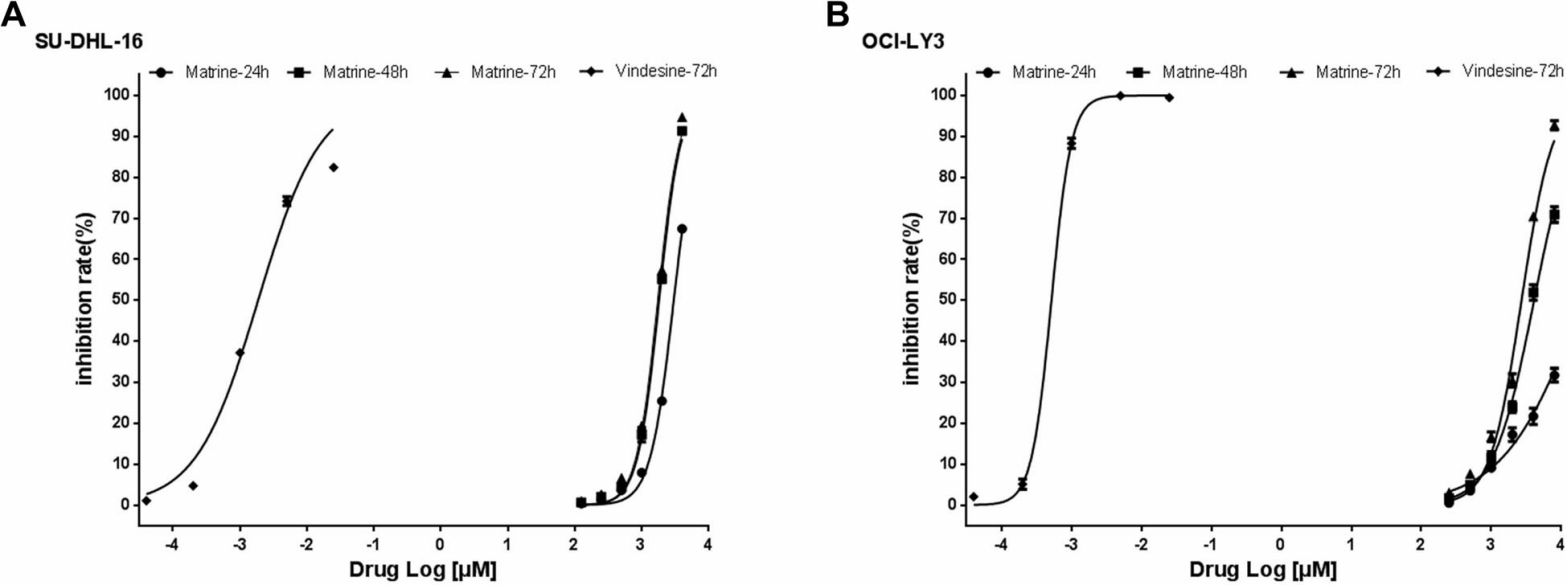
Anti-proliferation of matrine in DLBCL cells. **a**-**b** SU-DHL-16 and OCI-LY3 cells were treated with matrine and vindesine at different concentrations for different times. The total viable cells were determined by CCK-8 assay. Analyses in triplicates

Human DLBCL cell lines SU-DHL-16 and OCI-LY3 were maintained in exponential phase with good morphology (Supplementary Figure 1). The cell viability was determined to evaluate the percentage inhibition rate and IC_50_ of matrine in SU-DHL-16 and OCI-LY3 cells. As shown in Fig. 1a, matrine displayed anti-proliferative activity in SU-DHL-16 cells in a dose dependent and time dependent manner. The IC_50_ values of matrine in SU-DHL-16 cells were 2.98 ± 0.03, 1.76 ± 0.05 and 1.54 ± 0.04 mM, respectively, for 24 h, 48 h and 72 h while that of vindesine in SU-DHL-16 cells for 72 h was 1.33 ± 0.02 nM. As shown in Fig. 1b, matrine also exhibited anti-proliferative activity in OCI-LY3 cells in a dose and time dependent manner. The IC_50_ values of matrine in OCI-LY3 cells were 20.04 ± 0.05, 4.10 ± 0.05 and 2.67 ± 0.04 mM, respectively, for 24 h, 48 h and 72 h while that of vindesine in OCI-LY3 cells for 72 h was 0.52 ± 0.02 nM. Although exposure to matrine for a longer time than 48 h was found to be more potent in inhibiting SU-DHL-16 and OCI-LY3 cell viability, it inclined to result in increased frequency of necrotic cells, so we chose the treatment with matrine for no longer than 48 h for the following experiments in DLBCL cells.

### Matrine induces apoptosis

**Fig. 2:**
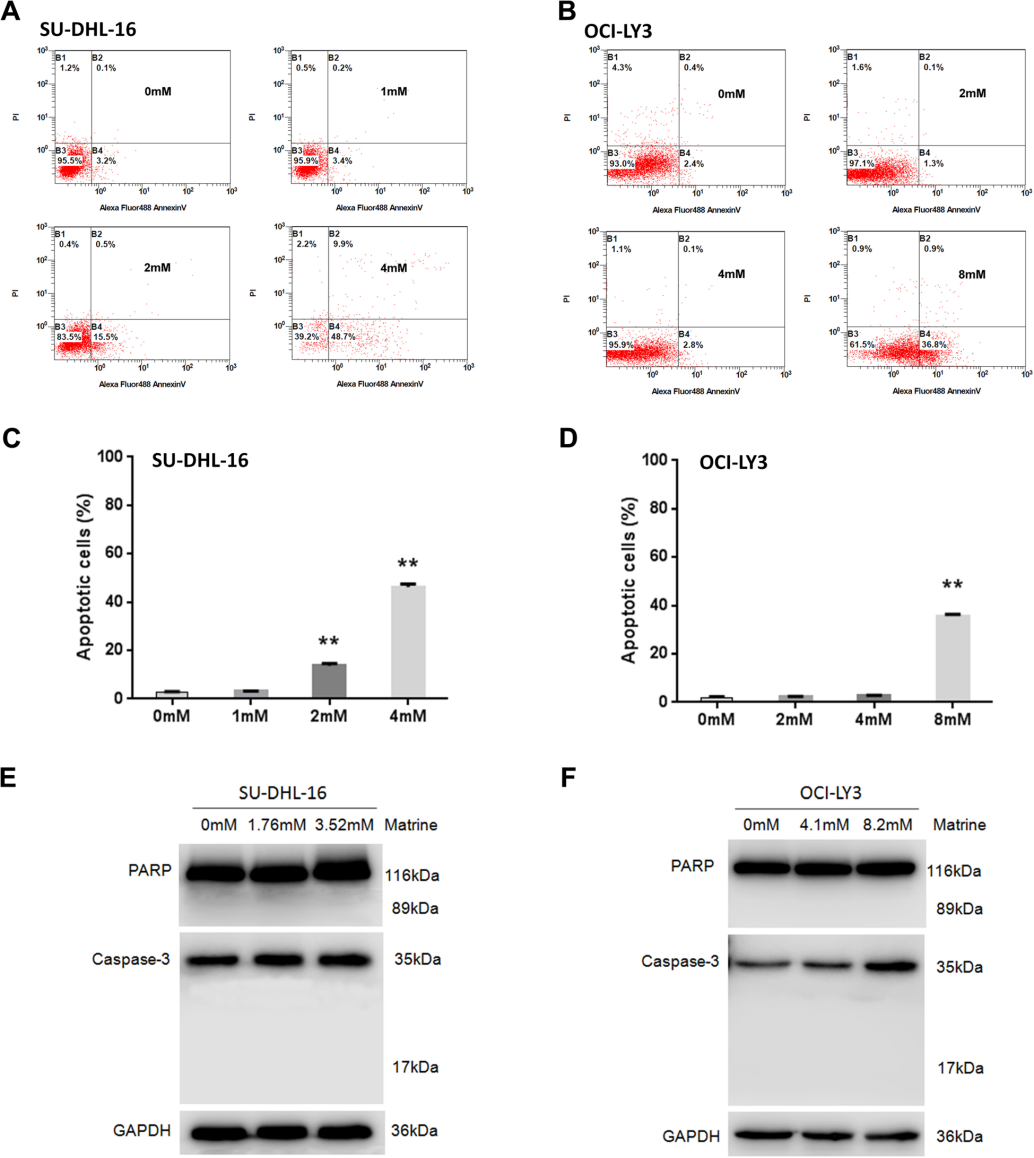
Apoptosis induction of matrine in DLBCL cells through caspase-independent pathway. **a**-**b** SU-DHL-16 and OCI-LY3 cells were treated by matrine at different concentrations for 48 h and then determined for apoptotic cells by annexin V and PI staining with flow cytometry. **c**-**d** Percent of apoptotic cells induced by matrine at various concentrations. **e**-**f** SU-DHL-16 and OCI-LY3 cells were treated with different concentrations of matrine for 48 h, followed by western blot. GAPDH was used as a loading control. Full-length blots are presented in Supplementary Figure 2. Analyses in triplicates. (**p < 0.01 compare to 0 mM group)

To explore whether the growth inhibition of DLBCL cells induced by matrine was caused by apoptosis, SU-DHL-16 and OCI-LY3 cells were treated with the increasing concentrations of matrine for 48 h, and the occurrence of apoptosis was determined by the annexin V and PI staining (Fig. 2a and b). Overall, the result in Fig. 2c showed an increase in the percentage of apoptotic cells from 2.8 ± 0.4% in untreated cells to 3.1 ± 0.3, 13.8 ± 1.5 and 46.2 ± 2.3% for 1, 2 and 4 mM matrine treatment to SU-DHL-16 cells, respectively. As shown in Fig. 2d, matrine induced an increase in the percentage of apoptotic cells from 2.0 ± 0.6% in untreated cells to 2.4 ± 0.2, 2.8 ± 0.3 and 35.6 ± 1.2% for 2, 4 and 8 mM matrine treatment to OCI-LY3 cells, respectively.

### Matrine regulates the expression of apoptosis-related proteins

To investigate the mechanism responsible for matrine mediated apoptosis, the apoptotic protein expressions were analyzed by western blot. Figure 2e and Supplementary Figures 2A - 2C showed the results of western blot for activated cleaved Caspase-3 (17 kDa) and cleaved PARP (89 kDa) in control cultures and cultures exposed to 1.76 mM (IC_50_ of 48 h) and 3.52 mM matrine for 48 h in SU-DHL-16 cells. Figure 2f and Supplementary Figures 2D - 2F showed the western blot results in control cultures and cultures exposed to 4.1 mM (IC_50_ of 48 h) and 8.2 mM matrine for 48 h in OCI-LY3 cells. Matrine treatment did not induce activated cleaved Caspase-3 and cleaved PARP in SU-DHL-16 and OCI-LY3 cells, which indicated that matrine induces apoptosis of DLBCL cells via caspase-independent pathway.

### Matrine induces cell cycle arrest at G_0_/G_1_ phase

**Fig. 3:**
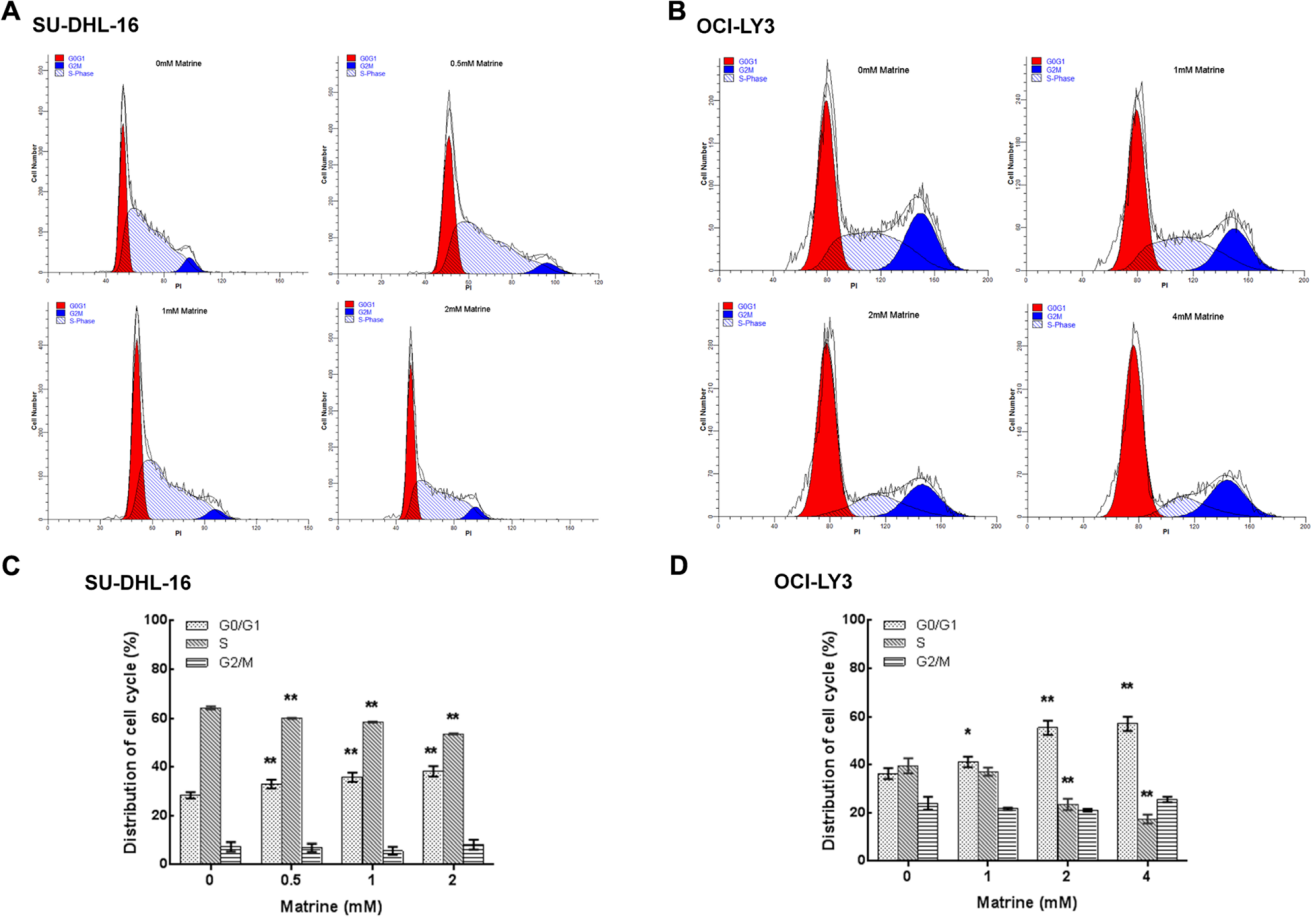
G_0_/G_1_ cell cycle arrest in matrine-treated DLBCL cells. **a**-**b** SU-DHL-16 and OCI-LY3 cells were exposed to matrine at different concentrations for 24 h and then stained with PI and analyzed for DNA content by flow cytometry. **c**-**d** Distribution of cell cycle percent induced by matrine at various concentrations. Analyses in triplicates. (*p < 0.05, **p < 0.01 compare to 0 mM group)

To further resolve the mechanism of anti-proliferative activity of matrine in DLBCL cells, cell cycle progression was examined by flow cytometry. As shown in Fig. 3, matrine induced the significant accumulation of cells in G_0_/G_1_ phase from 28.36 ± 1.28% before treatment to 33.03 ± 1.81, 35.82 ± 1.90 and 38.22 ± 2.15% after 0.5, 1 and 2 mM matrine treatment for 24 h in SU-DHL-16 cells or from 36.39 ± 2.25% before treatment to 41.20 ± 2.21, 55.42 ± 2.99 and 57.02 ± 3.03% after 1, 2 and 4 mM matrine treatment for 24 h in OCI-LY3 cells, respectively. This was accompanied by an apparent decrease of cells in S phase from 64.36 ± 0.62% before treatment to 60.07 ± 0.2, 58.47 ± 0.22 and 53.61 ± 0.19% after 0.5, 1 and 2 mM matrine treatment in SU-DHL-16 cells or from 39.58 ± 3.15% before treatment to 37.10 ± 1.70, 23.53 ± 2.37 and 17.42 ± 1.86% after 1, 2 and 4 mM matrine treatment in OCI-LY3 cells, respectively. The proportion in G_2_/M phase was not significantly changed after matrine treatment in both cells. These results clearly indicated that matrine induces G_0_/G_1_ cell cycle arrest in DLBCL cells.

### Matrine downregulates c-Myc expression through increased degradation

**Fig. 4:**
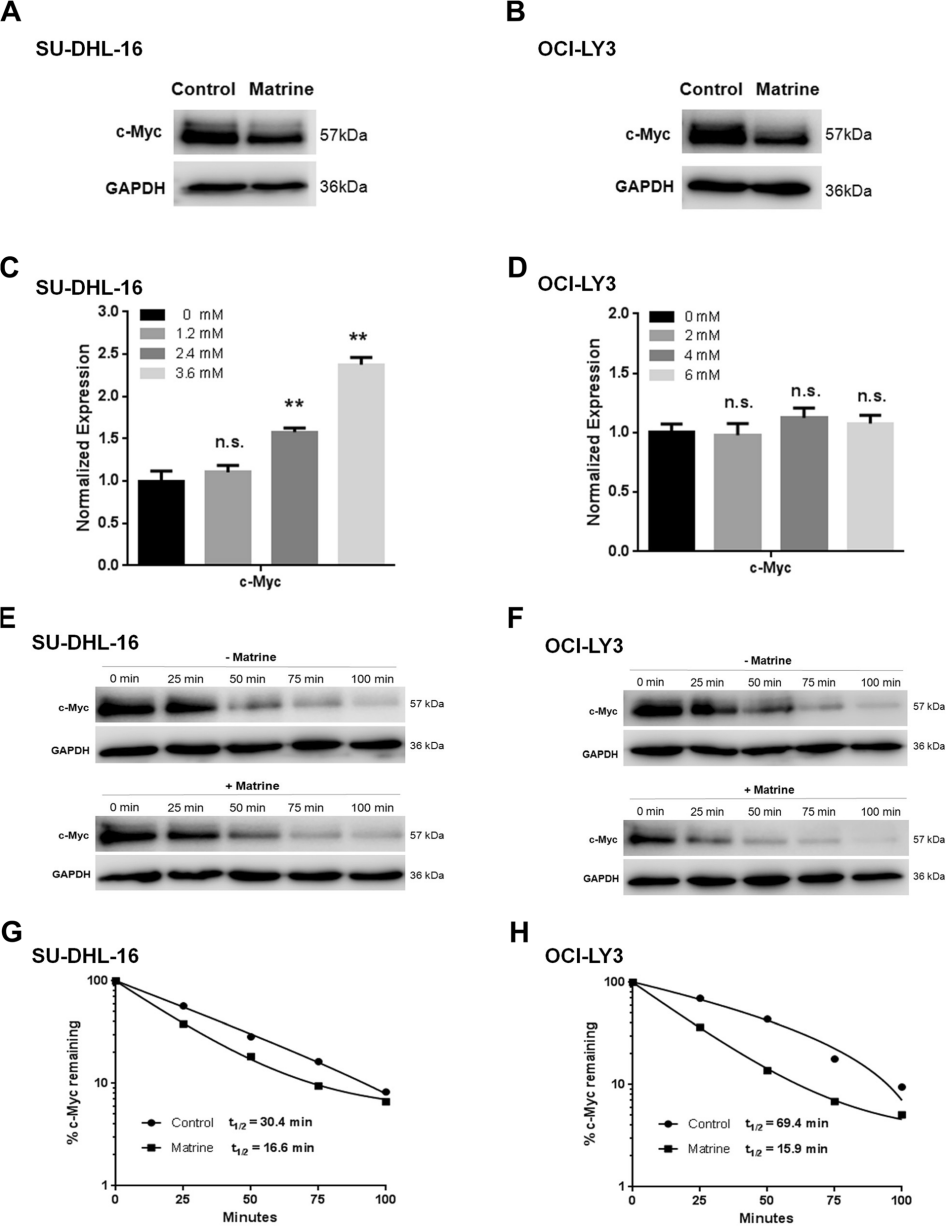
Reduced c-Myc protein expression by matrine in DLBCL cells. **a**-**b** SU-DHL-16 and OCI-LY3 cells were exposed to 1.76 mM and 4.1 mM matrine for 48 h, respectively, and c-Myc protein levels were measured by western blot. Full-length blots are presented in Supplementary Figure 3. **c**-**d** c-Myc mRNA levels in SU-DHL-16 and OCI-LY3 cells were determined by quantitative RT-PCR at 24 h after different concentrations of matrine treatment (n.s., not significant; **p < 0.01 compared to 0 mM group). **e**-**f** CHX chase assay for the half-life of c-Myc. SU-DHL-16 and OCI-LY3 cells were treated with or without 1.76 mM and 4.1 mM matrine for 12 h, respectively. Cells were then treated with CHX for the indicated minutes, and western blotting was performed. Full-length blots are presented in Supplementary Figure 4. **g**-**h** c-Myc levels were quantified relative to GAPDH levels and graphed as percent c-Myc protein remaining after CHX treatment. Half-lives of c-Myc were calculated from exponential line equations and shown for each treat. Analyses in triplicates

c-Myc expression is closely related to cell proliferation. The expression of c-Myc protein in DLBCL cells was then analyzed by western blot. SU-DHL-16 and OCI-LY3 cells were treated with matrine at 1.76 mM and 4.1 mM for 48 h, respectively. The levels of c-Myc protein in SU-DHL-16 and OCI-LY3 cells were significantly reduced with normalized relative expression of 0.601 ± 0.086 and 0.404 ± 0.046 after matrine treatment (Fig. 4a, b and Supplementary Figure 3), respectively. These results indicated that the growth inhibition induced by matrine in DLBCL cells is associated with the downregulation of c-Myc protein.

Transcription analysis of c-Myc gene was then processed. SU-DHL-16 and OCI-LY3 cells were incubated with increasing concentrations of matrine for 24 h. The qRT-PCR results showed that c-Myc mRNA levels were significantly increased after matrine treatment in a dose dependent manner in SU-DHL-16 cells (Fig. 4c), while c-Myc mRNA levels were not significantly changed in OCI-LY3 cells (Fig. 4d).

The degradation of c-Myc protein was also evaluated by cycloheximide chase assay. The translation inhibitor cycloheximide was put into the control and matrine-treated SU-DHL-16 and OCI-LY3 cells, and c-Myc protein levels were determined at different time points by western blot. Half-lives of c-Myc protein were then estimated by GraphPad Prism program. As shown in Figs. 4e-h and Supplementary Figure 4, c-Myc half-lives in matrine-treated SU-DHL-16 and OCI-LY3 cells were about 16.6 and 15.9 min, while those in the control SU-DHL-16 and OCI-LY3 cells were about 30.4 and 69.4 min, respectively. The results indicated that c-Myc degradation is promoted and the stability of c-Myc is decreased in matrine-treated DLBCL cells.

### Matrine reduces c-Myc phosphorylation at Ser62 through CaMKIIγ inhibition

**Fig. 5:**
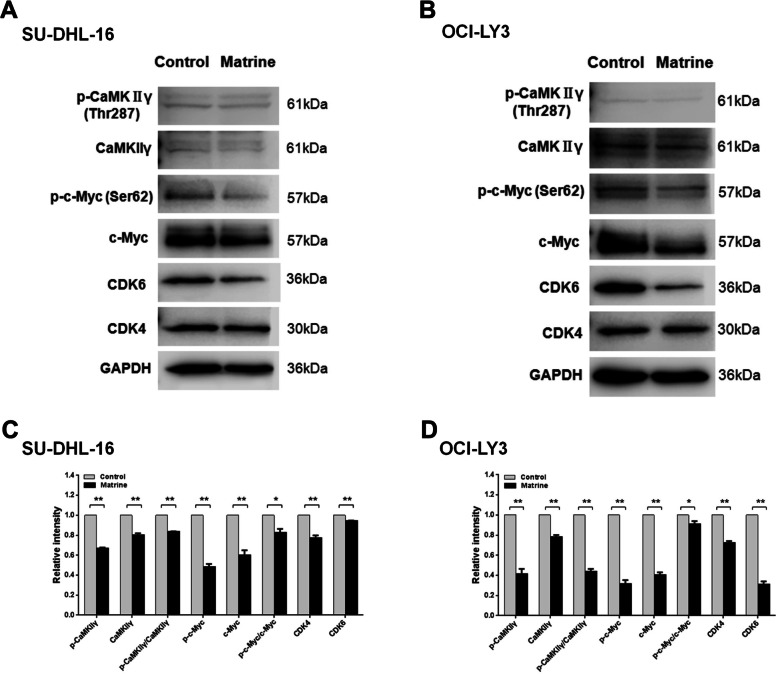
Matrine inhibited DLBCL cells via CaMKIIγ/c-Myc/CDK pathway. **a**-**b** SU-DHL-16 and OCI-LY3 cells were treated with 1.76 mM and 4.1 mM matrine for 48 h, respectively, and followed by western blot for p-CaMKIIγ (Thr287), CaMKIIγ, p-c-Myc (Ser62), c-Myc, CDK6 and CDK4 antibodies. GAPDH was used as loading control. Full-length blots are presented in Supplementary Figure 5. **c**-**d** The relative intensities of target proteins were normalized to those of loading control. Analyses in triplicates. (*p < 0.05, **p < 0.01)

c-Myc protein stability is tuned by two phosphorylation sites with opposite effects: threonine 58 phosphorylation promotes c-Myc degradation while serine 62 phosphorylation stabilizes c-Myc. The phosphorylation of c-Myc at Ser62 in DLBCL cells was then determined by western blot. SU-DHL-16 and OCI-LY3 cells were treated with matrine at 1.76 mM and 4.1 mM for 48 h, respectively. The levels of phospho-c-Myc (Ser62) in SU-DHL-16 and OCI-LY3 cells were significantly reduced after matrine treatment suggesting the stability of c-Myc is declined (Fig. 5 and Supplementary Figure 5).

CaMKIIγ is the protein kinase that phosphorylates c-Myc at Ser62. After binding Ca^2+^/calmodulin complex, autophosphorylation of CaMKIIγ at Thr287 results into activated stage. The phosphorylation of CaMKIIγ in matrine-treated DLBCL cells was then analyzed by western blot. As shown in Fig. 5 and Supplementary Figure 5, the levels of phospho-CaMKIIγ (Thr287) were also significantly reduced after matrine treatment. These results indicated that matrine reduces c-Myc phosphorylation at Ser62 by targeting CaMKIIγ.

### Matrine down-regulates CDK4/6 expression

c-Myc-induced cell proliferation is generally associated with activation of CDK4 and CDK6 to control G_1_/S phase progression. CDK4 and CDK6 are described as c-Myc target genes. The expressions of CDK4 and CDK6 in matrine-treated DLBCL cells were then analyzed by western blot. As shown in Fig. 5 and Supplementary Figure 5, the levels of CDK4 and CDK6 protein were also significantly decreased after matrine treatment. These results suggested that matrine reduces CDK4 and CDK6 expression through c-Myc inhibition.

### Over-expression of c-Myc rescues the growth of SU-DHL-16 cells inhibited by matrine

**Fig. 6:**
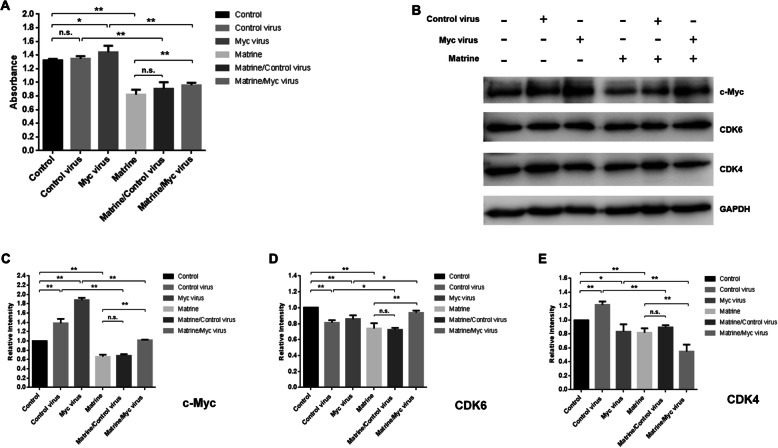
Salvage of matrine-induced growth inhibition rescued by ectopic expression of c-Myc. **a** SU-DHL-16 cells were treated with or without 1.76 mM matrine and recombinant c-Myc adenovirus or control adenovirus for 48 h. The total viable cells were determined by CCK-8 analysis. **b** Western blot for c-Myc, CDK6 and CDK4 antibodies. GAPDH was used as loading control. Full-length blots are presented in Supplementary Figure 6. **c**-**e** The relative intensities of target proteins were normalized to those of loading control. Analyses in triplicates. (n.s., not significant; *p < 0.05, **p < 0.01)

To further investigate the mechanism of growth inhibition induced by matrine in DLBCL cells, the recombinant adenovirus for c-Myc over-expression was used to explore the salvage effect in matrine-treated SU-DHL-16 cells by CCK-8 analysis. The results showed that c-Myc recombinant adenovirus infection significantly promoted the growth of SU-DHL-16 cells while the Control recombinant adenovirus did not affect the cell growth significantly (Fig. 6a). The cell growth inhibited by matrine was significantly rescued by the c-Myc recombinant adenovirus infection, not by the Control recombinant adenovirus infection, in SU-DHL-16 cells (Fig. 6a). Western blot confirmed the over-expression of c-Myc protein in c-Myc recombinant adenovirus infected SU-DHL-16 cells (Fig. 6b, c and Supplementary Figure 6). To determine whether CDK4 or CDK6 is a bona fide c-Myc target gene in SU-DHL-16 cells, the protein expressions of CDK4 and CDK6 were analyzed by western blot in SU-DHL-16 cells infected by c-Myc adenovirus or Control adenovirus with or without matrine treatment. The results clearly showed that the expression of CDK6, not CDK4, inhibited by matrine was significantly rescued by the c-Myc adenovirus infection, not by the Control adenovirus infection, in SU-DHL-16 cells (Fig. 6b, d, e and Supplementary Figure 6), which identified that CDK6 is a bona fide c-Myc target gene in SU-DHL-16 cells. These results verified that matrine inhibits the growth of SU-DHL-16 cells through CaMKIIγ-c-Myc-CDK6 pathway.

## Discussion

Matrine is a quinolizindine alkaloid with strong anticancer property towards various types of tumors. Han et al. reported that matrine inhibited human multiple myeloma RPMI8226 and U266 cells for 48 h with IC_50_ of 4.55 mM and 5.36 mM, respectively [13]. Ma et al. showed that matrine suppressed the growth of human chronic myeloid leukemia K562 cells with an IC_50_ of 2 mM for 48 h treatment [11]. Our previous study showed that matrine inhibited human NKTCL NK92 cells for 48 h with IC_50_ of 1.96 mM [14]. In current study, matrine inhibited the growth of DLBCL cell lines SU-DHL-16 and OCI-LY3 cells in a dose and time dependent manner. It displayed anti-proliferative activities against SU-DHL-16 and OCI-LY3 cells at 48 h with IC_50_ of 1.76 ± 0.05 mM and 4.10 ± 0.05 mM, respectively. Matrine was more effective for SU-DHL-16 cells than that for OCI-LY3 cells, which meant matrine is better for germinal center B-cell lymphoma subtype than that for active B-cell lymphoma subtype. Vindesine is an alkaloid for the clinic treatment of DLBCL [15]. It inhibited the growth of SU-DHL-16 and OCI-LY3 cells with IC_50_ of 1.33 ± 0.02 nM and 0.52 ± 0.02 nM for 72 h treatment, respectively. Considering the IC_50_ of matrine is much higher than that of vindesine, the influence of matrine on the normal lymphocytes is of much concern. Our previous study showed that matrine with lower than 2 mM did not significantly influence the proliferation of healthy human peripheral blood mononuclear cells (PBMCs) induced by PHA for 72 h [14]. Han et al. disclosed that lower concentrations of matrine (1, 2, 4, 6 mM) had no influence on the PBMCs proliferation and higher concentrations of matrine (8, 12, 20 mM) inhibited the proliferation of PBMCs for 72 h [13]. They also demonstrated that 2, 6, 12 and 20 mM matrine had no effects on the apoptosis of PBMCs for 48 h [13]. Our data and previous literature strongly support that matrine with lower than 6 mM has no harmful effects on the normal PBMCs, which will be beneficial for DLBCL patients.

Our previous study showed that matrine induced caspase-dependent apoptosis in NKTCL cells [14]. In present study, matrine induced apoptosis of DLBCL cell lines SU-DHL-16 and OCI-LY3 cells in a dose dependent manner from 2.8 ± 0.4% and 2.4 ± 0.2% to 46.2 ± 2.3% and 35.6 ± 1.2% after matrine treatment at different concentrations for 48 h, respectively (Fig. 2). It is interesting to notice that apoptosis caused by 2 mM matrine in SU-DHL-16 cells was less than 14% even though this dose was more than IC_50_ (1.76 mM). The apoptosis induced by 4 mM matrine in OCI-LY3 cells was near to the control group although this dose was close to IC_50_ (4.1 mM). These data suggests matrine does not preferentially induce apoptosis. The growth inhibition of DLBCL cells induced by matrine was partially caused by apoptosis. Furthermore, matrine did not induce the products of activated cleaved-Caspase-3 and cleaved PARP even with 2 fold of IC_50_ in DLBCL cells (Fig. 2). The execution of apoptosis comprises both caspase-dependent and caspase-independent pathways. Apoptosis inducing factor (AIF) was critical for caspase-independent cell death by direct interaction with DNA [16]. Our finding indicated that matrine induces apoptosis of DLBCL cells through the activation of caspase-independent pathway. The detailed molecular mechanism of caspase-independent cell death induced by matrine in DLBCL cells needs to be addressed in the future.

Deregulation of cell cycle is an important process in malignant transformation. Zhao et al. reported that matrine inhibited the growth of human retinoblastoma cells and induced cell cycle arrest at G_0_/G_1_ phase [17]. Jin et al. showed that matrine suppressed melanoma M21 cells proliferation by promoting G_0_/G_1_ cell cycle arrest [18]. Ma et al. reported that matrine inhibited human chronic myeloid leukemia K562 cells proliferation through promoting G_0_/G_1_ arrest [11]. In current study, matrine induced the significant G_0_/G_1_ phase arrest from 28.36 ± 1.28% to 33.03 ± 1.81, 35.82 ± 1.90 and 38.22 ± 2.15% after 0, 0.5, 1 and 2 mM matrine treatment for 24 h in SU-DHL-16 cells or from 36.39 ± 2.25% to 41.20 ± 2.21, 55.42 ± 2.99 and 57.02 ± 3.03% after 0, 1, 2 and 4 mM matrine treatment for 24 h in OCI-LY3 cells, respectively (Fig. 3). Our data showed that matrine preferentially induces G_0_/G_1_ cell cycle arrest, not apoptosis, to inhibit the growth of DLBCL cells. G_0_/G_1_ cell cycle arrest is the major mechanism for the matrine-induced growth inhibition in DLBCL cells. Based on the classical cell cycle model, in the early G_1_ phase, mitogenic signals are first received and integrated by the expression of cyclin D that preferentially binds to and activates CDK4 and CDK6 [19]. Our data showed that the expressions of CDK4 and CDK6 protein were significantly inhibited after matrine treatment in DLBCL cells (Fig. 5). These results indicated that matrine induces G_0_/G_1_ cell cycle arrest mostly through CDK4/6 inhibition in DLBCL cells.

c-Myc levels tightly correlate with cell proliferation. A major role for c-Myc in the proliferation of normal cells is to promote progression through G_1_ and into S phase of the cell cycle. A systematic study in 23 cell lines with short-hairpin-mediated depletion of c-Myc showed that arrest occurred at G_0_/G_1_ phase in normal cells and some tumor-derived cell lines [20]. c-Myc genetic alternations are the characterized events in DLBCL, which confer a more aggressive clinical behavior with dismal prognosis [8]. Our previous data showed that matrine inhibited the mRNA and protein expression of c-Myc in NKTCL NK92 cells [14]. Our present data showed that c-Myc protein expression was inhibited by matrine in DLBCL cells while the gene transcription of c-Myc was not suppressed (Fig. 4). The degradation of c-Myc protein was accelerated by matrine treatment in DLBCL cells. c-Myc protein half-lives were much shorter after exposure to matrine in SU-DHL-16 and OCI-LY3 cells (Fig. 4), which meant the stability of c-Myc protein in matrine-treated DLBCL cells was decreased. c-Myc protein stability is regulated by Thr58 phosphorylation which promotes c-Myc degradation, and Ser62 phosphorylation which stabilizes c-Myc [21]. Our data showed that the phosphorylation of c-Myc at Ser62 in DLBCL cells was significantly reduced after matrine treatment (Fig. 5), which confirmed that matrine decreases Ser62 phosphorylation of c-Myc to accelerate the degradation of c-Myc protein in DLBCL cells. The ectopic expression of c-Myc rescued the growth of matrine-treated SU-DHL-16 cells by recombinant adenovirus infection (Fig. 6), which verified that matrine inhibits the growth of SU-DHL-16 cells by c-Myc pathway.

c-Myc-induced cell proliferation is related to the increase of CDK4 and CDK6 activities to regulate G_1_/S progression [19]. CDK4 and CDK6 were listed as transcriptional targets of c-Myc. Hermeking et al. reported that c-Myc induced a rapid increase of CDK4 mRNA levels through four highly conserved c-Myc binding sites within the CDK4 promoter among different cell models. Cell-cycle progression is delayed in c-Myc-inactivated RAT1 fibroblast cells, and this delay was associated with a defect in induction of CDK4 protein [22]. Li et al. showed that c-Myc bound to CDK6 promoter in ChIP-on-chip analysis in the Burkitt’s lymphoma Daudi cells [23]. Zhang et al. reported that c-Myc promoted CDK6 expression through the repression of miR-29 in mantle cell lymphoma [24]. Our data showed that the expressions of CDK4 and CDK6 protein were significantly inhibited after matrine treatment in DLBCL cells (Fig. 5). The ectopic expression of c-Myc rescued the expression of CDK6, not CDK4, in the matrine treated SU-DHL-16 cells (Fig. 6), which identified that CDK6 is a bona fide c-Myc target gene in SU-DHL-16 cells. Present study indicated that matrine inhibits CDK4 protein expression independent of c-Myc in SU-DHL-16 cells. The detailed molecular mechanism of CDK4 inhibition induced by matrine in DLBCL cells needs to be addressed in the future. These results supported that matrine inhibits the growth of SU-DHL-16 cells partly through c-Myc-CDK6 pathway.

CaMKIIγ was validated to be one of 102 potential genes involved in a synthetic lethal interaction with c-Myc [25]. CaMKIIγ phosphorylated Ser62 of c-Myc and increased the stability of c-Myc in T cell lymphoma. Inhibition of CaMKIIγ decreased the protein levels of c-Myc and suppressed the growth of T cell lymphoma H9 cells in vitro, whereas overexpression of CaMKIIγ significantly increased the c-Myc transcriptional activity [26]. Our previous study showed that matrine inhibited the growth of NKTCL cells by modulating CaMKIIγ-c-Myc pathway [14]. Present study showed that a positive correlation between CaMKIIγ and p-c-Myc (Ser62)/c-Myc was observed in DLBCL cells. The levels of phospho-c-Myc (Ser62) and CaMKIIγ in SU-DHL-16 and OCI-LY3 cells were together remarkably reduced after matrine treatment (Fig. 5). Our findings indicated that matrine suppresses cell growth of DLBCL by regulating CaMKIIγ/c-Myc pathway. The specific functions of CaMKIIγ in DLBCL are not fully known, our data support that CaMKIIγ inhibition may be a great way to treat c-Myc-driven DLBCL.

Indirect inhibition of c-Myc represents a great opportunity to cure associated cancers. The first small molecule bromodomain inhibitor, JQ1, inhibited the c-Myc function and its target genes in DLBCL [27]. Alkaloids, such as matrine, berberine and vindesine, are strong therapeutic agents for cancers. Ma et al. reported that one of berberine derivations, quinolino-benzo-[5,6]-dihydroisoquindolium compound, inhibited the c-Myc transcription by selectively binding G-quadruplex c-Myc DNA in leukemia cell line HL60 [28]. Small molecule analog of berbamine, tosyl chloride-berbamine, inhibited CaMKIIγ expression to decrease c-Myc protein in c-Myc-positive leukemia cells [29]. Our data confirmed that matrine accelerated c-Myc protein degradation via CaMKIIγ inhibition in DLBCL cells. CaMKIIγ/Myc axis represents a promising target in Myc-mediated DLBCL. Matrine will be helpful for those c-Myc-driven DLBCL patients.

Limitations of this study focus on the DLBCL cell lines. As matrine induces ROS, apoptosis and autophagy, present study showed that the effects of matrine on DLBCL cells were mainly dependent on the growth inhibition via CaMKIIγ/c-Myc/CDK6 pathway. The effects of matrine on primary DLBCL cells and DLBCL in vivo need to be investigated in the future.

## Conclusions

In summary, present study showed that matrine suppresses cell growth through G_0_/G_1_ cell cycle arrest and caspase-independent apoptosis in DLBCL cells. Our results have demonstrated for the first time that the mechanism of matrine suppressing the cell growth of DLBCL is the inhibition of CaMKIIγ/c-Myc/CDK6 signaling pathway. There is great demand for treating c-Myc-driven DLBCL. It is reasonable that matrine may be useful as a complementary medicine for DLBCL.

## Supplementary Information

**Additional file 1: Supplementary Figure 1.** The morphology of SU-DHL-16 and OCI-LY3 cells. (A) SU-DHL-16 (400×). (B) OCI-LY3 (400×). Scale bar at the bottom right represents 100 μm. **Supplementary Figure 2.** Matrine induced the expression of apoptosis-related proteins in DLBCL cells. SU-DHL-16 cells (2.5×10^6^) were treated with 1.76 mM and 3.52 mM matrine for 48 h and OCI-LY3 cells (2.5×10^6^) were treated with 4.1 mM and 8.2 mM matrine for 48 h. Cells treated without matrine were used as control. Western blot was performed. (A) Representative WB result of Caspase-3 and cleaved Caspase-3 in SU-DHL-16 cells. (B) Representative WB result of PARP and cleaved PARP in SU-DHL-16 cells. (C) Representative WB result of GAPDH, the loading control for A and B. (D) Representative WB result of Caspase-3 and cleaved Caspase-3 in OCI-LY3 cells. (E) Representative WB result of PARP and cleaved PARP in OCI-LY3 cells. (F) Representative WB result of GAPDH, the loading control for D and E. **Supplementary Figure 3.** Matrine decreased the expression of c-Myc protein in DLBCL cells. SU-DHL-16 and OCI-LY3 cells (2.5×10^6^) were treated with matrine at 1.76 mM and 4.1 mM for 48 h, respectively, and followed by western blot. Cells treated without matrine were used as control. (A) Representative WB result of c-Myc in SU-DHL-16 cells. (B) WB result of GAPDH, the loading control for A. (C) Representative WB result of c-Myc in OCI-LY3 cells. (D) WB result of GAPDH, the loading control for C. **Supplementary Figure 4.** Matrine promoted c-Myc protein degradation in DLBCL cells. Cycloheximide chase assay was used for the half-life of c-Myc protein. SU-DHL-16 and OCI-LY3 cells (1.25 × 10^6^) were treated with 1.76 mM and 4.1 mM matrine for 12 h, respectively. Cells were then treated with cycloheximide (100 μg/mL) for the indicated minutes, and western blotting was performed. Cells treated without matrine were used as control. (A) Representative WB results of c-Myc and GAPDH in the control and matrine-treated SU-DHL-16 cells, respectively. (B) Representative WB results of c-Myc and GAPDH in the control and matrine-treated OCI-LY3 cells, respectively. **Supplementary Figure 5.** Matrine inhibited the growth of DLBCL cells through CaMKIIγ/c-Myc/CDK pathway. SU-DHL-16 and OCI-LY3 cells (2.5×10^6^) were treated with matrine at 1.76 mM and 4.1 mM for 48 h, respectively, and followed by western blot. Cells treated without matrine were used as control. (A) Representative WB result of p-CaMKIIγ(Thr287) in SU-DHL-16 cells. (B) Representative WB result of CaMKIIγ in SU-DHL-16 cells. (C) Representative WB result of p-c-Myc (Ser62) in SU-DHL-16 cells. (D) Representative WB result of c-Myc in SU-DHL-16 cells. (E) Representative WB result of CDK6 in SU-DHL-16 cells. (F) Representative WB result of CDK4 in SU-DHL-16 cells. (G) WB result of GAPDH, the loading control for A, B, C, D, E and F. (H) Representative WB result of p-CaMKIIγ(Thr287) in OCI-LY3 cells. (I) Representative WB result of CaMKIIγ in OCI-LY3 cells. (J) Representative WB result of p-c-Myc (Ser62) in OCI-LY3 cells. (K) Representative WB result of c-Myc in OCI-LY3 cells. (L) Representative WB result of CDK6 in OCI-LY3 cells. (M) Representative WB result of CDK4 in OCI-LY3 cells. (N) WB result of GAPDH, the loading control for H, I, J, K, L and M. **Supplementary Figure 6.** Salvage of matrine-induced growth inhibition rescued by ectopic expression of c-Myc. SU-DHL-16 cells (1 × 10^6^) were treated with or without 1.76 mM matrine and recombinant c-Myc adenovirus or control adenovirus for 48 h, followed by western blot. Cells treated without matrine and adenovirus were used as control. (A) Representative WB result of c-Myc. (B) Representative WB result of CDK6. (C) Representative WB result of CDK4. (D) WB result of GAPDH, the loading control for A, B and C.

## Data Availability

All data generated or analysed during this study are included in this published article and its supplementary information files.

## Abbreviations

CaMKIIγ: Ca^2+^/calmodulin-dependent protein kinase II γ
CDK: Cyclin-dependent protein kinase
CHX: Cycloheximide
DLBCL: Diffuse large B-cell lymphoma
IC_50_: Half maximal inhibitory concentration
NKTCL: NK/T-cell lymphoma

## References

[1] Swerdlow SH, Campo E, Harris NL. WHO Classifification of Tumours of Haematopoietic and lymphoid tissues. Revised 4th ed. Lyon: IARC Press; 2017.

[2] Liu Y, Barta SK (2019). Diffuse large B-cell lymphoma: 2019 update on diagnosis, risk stratification, and treatment. Am J Hematol.

[3] Sarkozy C, Sehn LH (2018). Management of relapsed/refractory DLBCL. Best Pract Res Clin Haematol.

[4] Miao Y, Medeiros LJ, Li Y, Li J, Young KH (2019). Genetic alterations and their clinical implications in DLBCL. Nat Rev Clin Oncol.

[5] Baluapuri A, Wolf E, Eilers M (2020). Target gene-independent functions of MYC oncoproteins. Nat Rev Mol Cell Biol.

[6] Allen-Petersen BL, Sears RC (2019). Mission possible: advances in MYC therapeutic targeting in Cancer. BioDrugs.

[7] Sesques P, Johnson NA (2017). Approach to the diagnosis and treatment of high-grade B-cell lymphomas with MYC and BCL2 and/or BCL6 rearrangements. Blood.

[8] Xia Y, Zhang X (2020). The Spectrum of MYC alterations in diffuse large B-cell lymphoma. Acta Haematol.

[9] Xiang Y, Guo Z, Zhu P, Chen J, Huang Y (2019). Traditional Chinese medicine as a cancer treatment: modern perspectives of ancient but advanced science. Cancer Med.

[10] You L, Yang C, Du Y, Wang W, Sun M, Liu J, et al. A systematic review of the pharmacology, Toxicology and Pharmacokinetics of Matrine. Front Pharmacol. 2020;11:01067. 10.3389/fphar.2020.01067 PMC7526649 33041782

[11] Ma L, Zhu Z, Jiang L, Sun X, Lu X, Zhou M, et al. Matrine suppresses cell growth of human chronic myeloid leukemia cells via its inhibition of the interleukin-6/Janus activated kinase/signal transducer and activator of transcription 3 signaling cohort. Leuk Lymphoma. 2015;56(10):2923–30. 10.3109/10428194.2015.1007507. 10.3109/10428194.2015.1007507 25629992

[12] Wu D, Shao K, Zhou Q, Sun J, Wang Z, Yan F, et al. Autophagy and ubiquitin-mediated Proteolytic degradation of PML/Rarα fusion protein in Matrine-induced differentiation sensitivity recovery of ATRA-resistant APL (NB4-LR1) cells: in vitro and in vivo studies. Cell Physiol Biochem. 2018;48(6):2286–301. 10.1159/000492646. 10.1159/000492646 30114705

[13] Han Y, Zhang S, Wu J, Yu K, Zhang Y, Yin L, et al. Matrine induces apoptosis of human multiple myeloma cells via activation of the mitochondrial pathway. Leuk Lymphoma. 2010;51:1337–46. 10.3109/10428194.2010.488708 20528251

[14] Gu J, Zhang Y, Wang X, Xiang J, Deng S, Wu D, et al. Matrine inhibits the growth of natural killer/T-cell lymphoma cells by modulating CaMKIIγ-c-Myc signaling pathway. BMC Complement Med Ther. 2020;20(1):214. 10.1186/s12906-020-03006-2. 10.1186/s12906-020-03006-2 PMC7346655 32641029

[15] Martino E, Casamassima G, Castiglione S, Cellupica E, Pantalone S, Papagni F, et al. Vinca alkaloids and analogues as anti-cancer agents: looking back, peering ahead. Bioorg Med Chem Lett. 2018;28(17):2816–26. 10.1016/j.bmcl.2018.06.044. 10.1016/j.bmcl.2018.06.044 30122223

[16] Bano D, Prehn JHM (2018). Apoptosis-inducing factor (AIF) in physiology and disease: the tale of a repented natural born killer. EBioMedicine.

[17] Zhao B, Li B, Bai S, Shen L, Ren R, Jonas JB, et al. Effects of matrine on proliferation and apoptosis of cultured retinoblastoma cells. Graefes Arch Clin Exp Ophthalmol. 2012;250(6):897–905. 10.1007/s00417-011-1751-4. 10.1007/s00417-011-1751-4 21866335

[18] Jin H, Sun Y, Wang S, Cheng X (2013). Matrine activates PTEN to induce growth inhibition and apoptosis in V600EBRAF harboring melanoma cells. Int J Mol Sci.

[19] Bretones G, Delgado MD, León J (2015). Myc and cell cycle control. Biochim Biophys Acta.

[20] Wang H, Mannava S, Grachtchouk V, Zhuang D, Soengas MS, Gudkov AV, et al. C-Myc depletion inhibits proliferation of human tumor cells at various stages of the cell cycle. Oncogene. 2008;27(13):1905–15. 10.1038/sj.onc.1210823. 10.1038/sj.onc.1210823 PMC3144565 17906696

[21] Sears R, Nuckolls F, Haura E, Taya Y, Tamai K, Nevins JR (2000). Multiple Ras-dependent phosphorylation pathways regulate Myc protein stability. Genes Dev.

[22] Hermeking H, Rago C, Schuhmacher M, Li Q, Barrett JF, Obaya AJ, et al. Identification of CDK4 as a target of c-MYC. Proc Natl Acad Sci U S A. 2000;97(5):2229–34. 10.1073/pnas.050586197. 10.1073/pnas.050586197 PMC15783 10688915

[23] Li Z, Van Calcar S, Qu C, Cavenee WK, Zhang MQ, Ren B. A global transcriptional regulatory role for c-Myc in Burkitt's lymphoma cells. Proc Natl Acad Sci U S A. 2003;100(14):8164–9. 10.1073/pnas.1332764100. 10.1073/pnas.1332764100 PMC166200 12808131

[24] Zhang X, Zhao X, Fiskus W, Lin J, Lwin T, Rao R, et al. Coordinated silencing of MYC- mediated miR-29 by HDAC3 and EZH2 as a therapeutic target of histone modification in aggressive B-cell lymphomas. Cancer Cell. 2012;22(4):506–23. 10.1016/j.ccr.2012.09.003. 10.1016/j.ccr.2012.09.003 PMC3973134 23079660

[25] Toyoshima M, Howie HL, Imakura M, Walsh RM, Annis JE, Chang AN, et al. Functional genomics identififies therapeutic targets for MYC-driven cancer. Proc Natl Acad Sci U S A. 2012;109(24):9545–50. 10.1073/pnas.1121119109. 10.1073/pnas.1121119109 PMC3386069 22623531

[26] Gu Y, Zhang J, Ma X, Kim BW, Wang H, Li J, et al. Stabilization of the c-Myc protein by CAMKIIgamma promotes T cell lymphoma. Cancer Cell. 2017;32(1):115–28. 10.1016/j.ccell.2017.06.001. 10.1016/j.ccell.2017.06.001 PMC5552197 28697340

[27] Chapuy B, McKeown MR, Lin CY, Monti S, Roemer MG, Qi J, et al. Discovery and characterization of super-enhancer-associated dependencies in diffuse large B cell lymphoma. Cancer Cell. 2013;24(6):777–90. 10.1016/j.ccr.2013.11.003. 10.1016/j.ccr.2013.11.003 PMC4018722 24332044

[28] Ma Y, Ou TM, Tan JH, Hou JQ, Huang SL, Gu LQ, et al. Quinolino-benzo-[5, 6]-dihydroisoquindolium compounds derived from berberine: a new class of highly selective ligands for G-quadruplex DNA in c-myc oncogene. Eur J Med Chem. 2011;46(5):1906–13. 10.1016/j.ejmech.2011.02.020. 10.1016/j.ejmech.2011.02.020 21392861

[29] Yu Q, Wang P, Yang L, Wu Z, Li S, Xu Y, et al. Novel synthetic tosyl chloride-berbamine regresses lethal MYC-positive leukemia by targeting CaMKIIγ/Myc axis. Biomed Pharmacother. 2019;117:109134. 10.1016/j.biopha.2019.109134. 10.1016/j.biopha.2019.109134 31247466

