# Supplementary material for: Matrine suppresses cell growth of diffuse large B-cell lymphoma via inhibiting CaMKIIγ/c-Myc/CDK6 signaling pathway
Source: BMC Complement Med Ther. 2021 Jun 4;21:163. doi: 10.1186/s12906-021-03315-0 (PMC8178855; doi:10.1186/s12906-021-03315-0)
Supplement: Supplementary file 1 — Additional file 1: Supplementary Figure 1. The morphology of SU-DHL-16 and OCI-LY3 cells. (A) SU-DHL-16 (400×). (B) OCI-LY3 (400×). Scale bar at the bottom right represents 100 μm. Supplementary Figure 2. Matrine induced the expression of apoptosis-related proteins in DLBCL cells. SU-DHL-16 cells (2.5×10^6^) were treated with 1.76 mM and 3.52 mM matrine for 48 h and OCI-LY3 cells (2.5×10^6^) were treated with 4.1 mM and 8.2 mM matrine for 48 h. Cells treated without matrine were used as control. Western blot was performed. (A) Representative WB result of Caspase-3 and cleaved Caspase-3 in SU-DHL-16 cells. (B) Representative WB result of PARP and cleaved PARP in SU-DHL-16 cells. (C) Representative WB result of GAPDH, the loading control for A and B. (D) Representative WB result of Caspase-3 and cleaved Caspase-3 in OCI-LY3 cells. (E) Representative WB result of PARP and cleaved PARP in OCI-LY3 cells. (F) Representative WB result of GAPDH, the loading control for D and E. Supplementary Figure 3. Matrine decreased the expression of c-Myc protein in DLBCL cells. SU-DHL-16 and OCI-LY3 cells (2.5×10^6^) were treated with matrine at 1.76 mM and 4.1 mM for 48 h, respectively, and followed by western blot. Cells treated without matrine were used as control. (A) Representative WB result of c-Myc in SU-DHL-16 cells. (B) WB result of GAPDH, the loading control for A. (C) Representative WB result of c-Myc in OCI-LY3 cells. (D) WB result of GAPDH, the loading control for C. Supplementary Figure 4. Matrine promoted c-Myc protein degradation in DLBCL cells. Cycloheximide chase assay was used for the half-life of c-Myc protein. SU-DHL-16 and OCI-LY3 cells (1.25 × 10^6^) were treated with 1.76 mM and 4.1 mM matrine for 12 h, respectively. Cells were then treated with cycloheximide (100 μg/mL) for the indicated minutes, and western blotting was performed. Cells treated without matrine were used as control. (A) Representative WB results of c-Myc and GAPDH in the control and matrine-treated SU- [file 12906_2021_3315_MOESM1_ESM.docx]

**Matrine suppresses cell growth of diffuse large B-cell lymphoma via inhibiting CaMKIIγ/c-Myc/CDK6 signaling pathway**

Jianyou Gu^1,2,*^, Xiao Wang^1,2^, Ling Zhang^1^, Jingjing Xiang^1^, Jingya Li^1,2^, Zheng Chen^1,2^, Yu Zhang^1^, Junfa Chen^1^, Jianping Shen^1, *^

Supplementary Information:

Supplementary Figures

Supplementary Figure Legends

**Supplementary Figure 1**

**
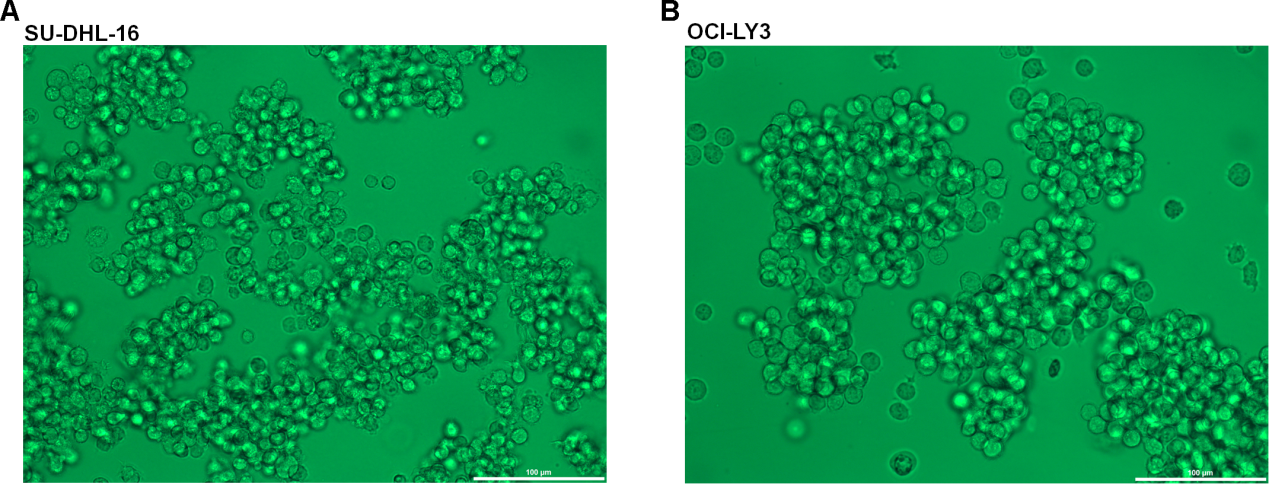
**

**Supplementary Figure 1.** The morphology of SU-DHL-16 and OCI-LY3 cells. (A) SU-DHL-16 (400×). (B) OCI-LY3 (400×). Scale bar at the bottom right represents 100 μm.

**Supplementary Figure 2**

**
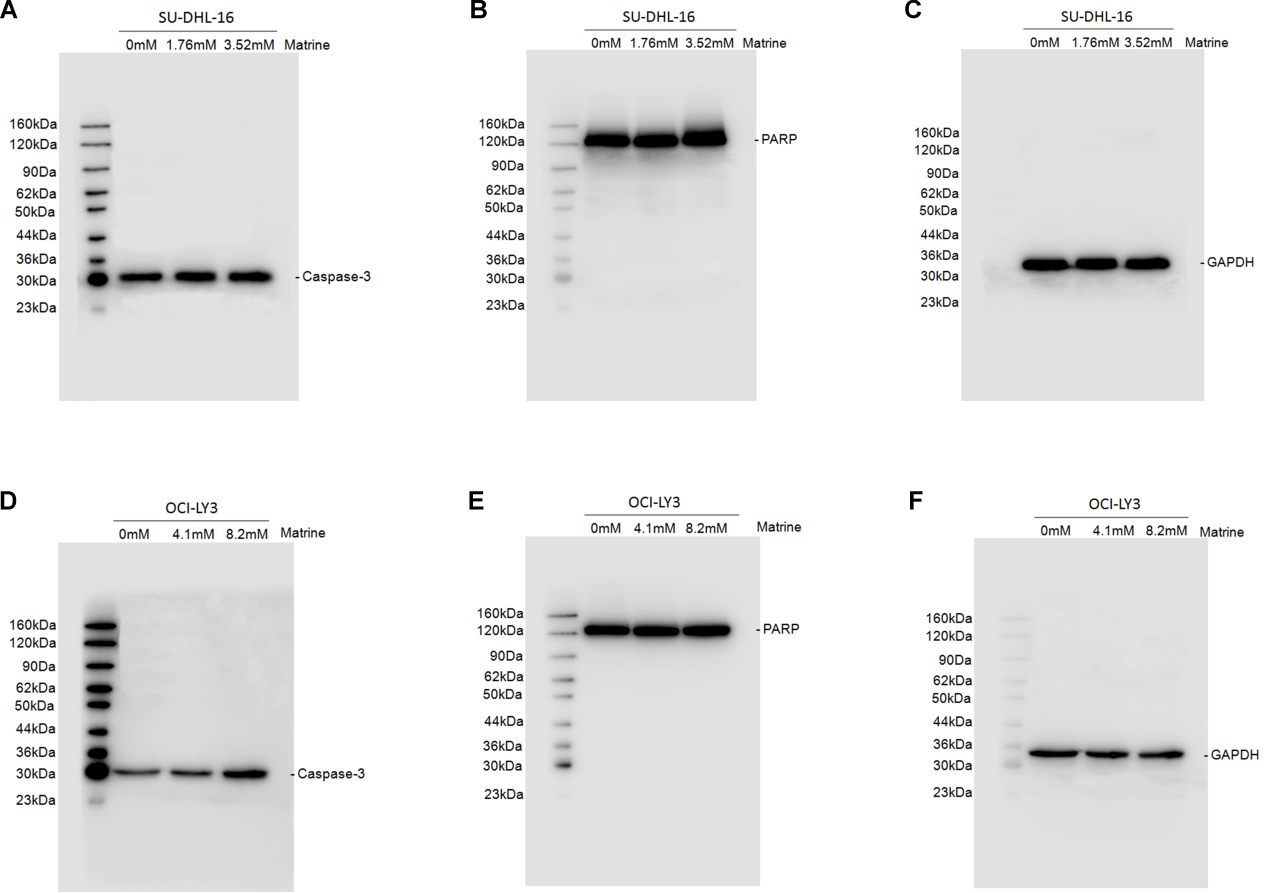
**

**Supplementary Figure 2.** Matrine induced the expression of apoptosis-related proteins in DLBCL cells. SU-DHL-16 cells (2.5×10^6^) were treated with 1.76 mM and 3.52 mM matrine for 48 h and OCI-LY3 cells (2.5×10^6^) were treated with 4.1 mM and 8.2 mM matrine for 48 h. Cells treated without matrine were used as control. Western blot was performed. (A) Representative WB result of Caspase-3 and cleaved Caspase-3 in SU-DHL-16 cells. (B) Representative WB result of PARP and cleaved PARP in SU-DHL-16 cells. (C) Representative WB result of GAPDH, the loading control for A and B. (D) Representative WB result of Caspase-3 and cleaved Caspase-3 in OCI-LY3 cells. (E) Representative WB result of PARP and cleaved PARP in OCI-LY3 cells. (F) Representative WB result of GAPDH, the loading control for D and E.

**Supplementary Figure 3**

**
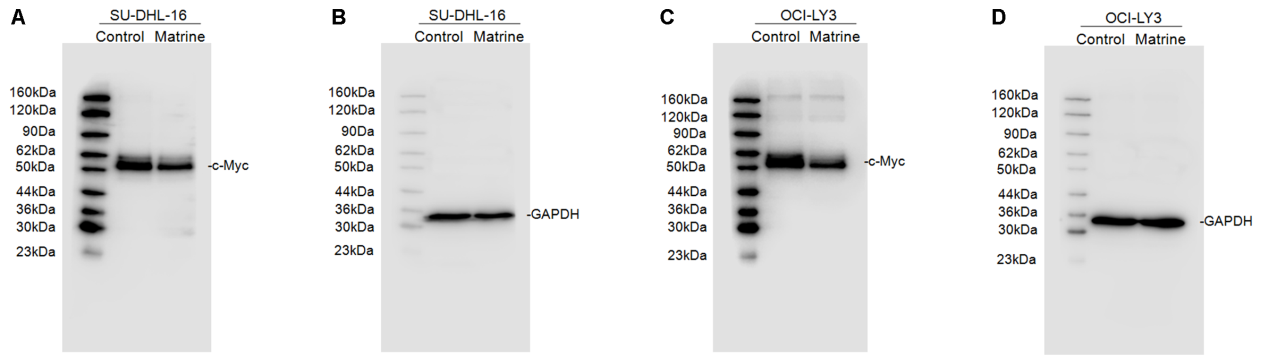
**

**Supplementary Figure 3**. Matrine decreased the expression of c-Myc protein in DLBCL cells. SU-DHL-16 and OCI-LY3 cells (2.5×10^6^) were treated with matrine at 1.76 mM and 4.1 mM for 48 h, respectively, and followed by western blot. Cells treated without matrine were used as control. (A) Representative WB result of c-Myc in SU-DHL-16 cells. (B) WB result of GAPDH, the loading control for A. (C) Representative WB result of c-Myc in OCI-LY3 cells. (D) WB result of GAPDH, the loading control for C.

**Supplementary Figure 4**


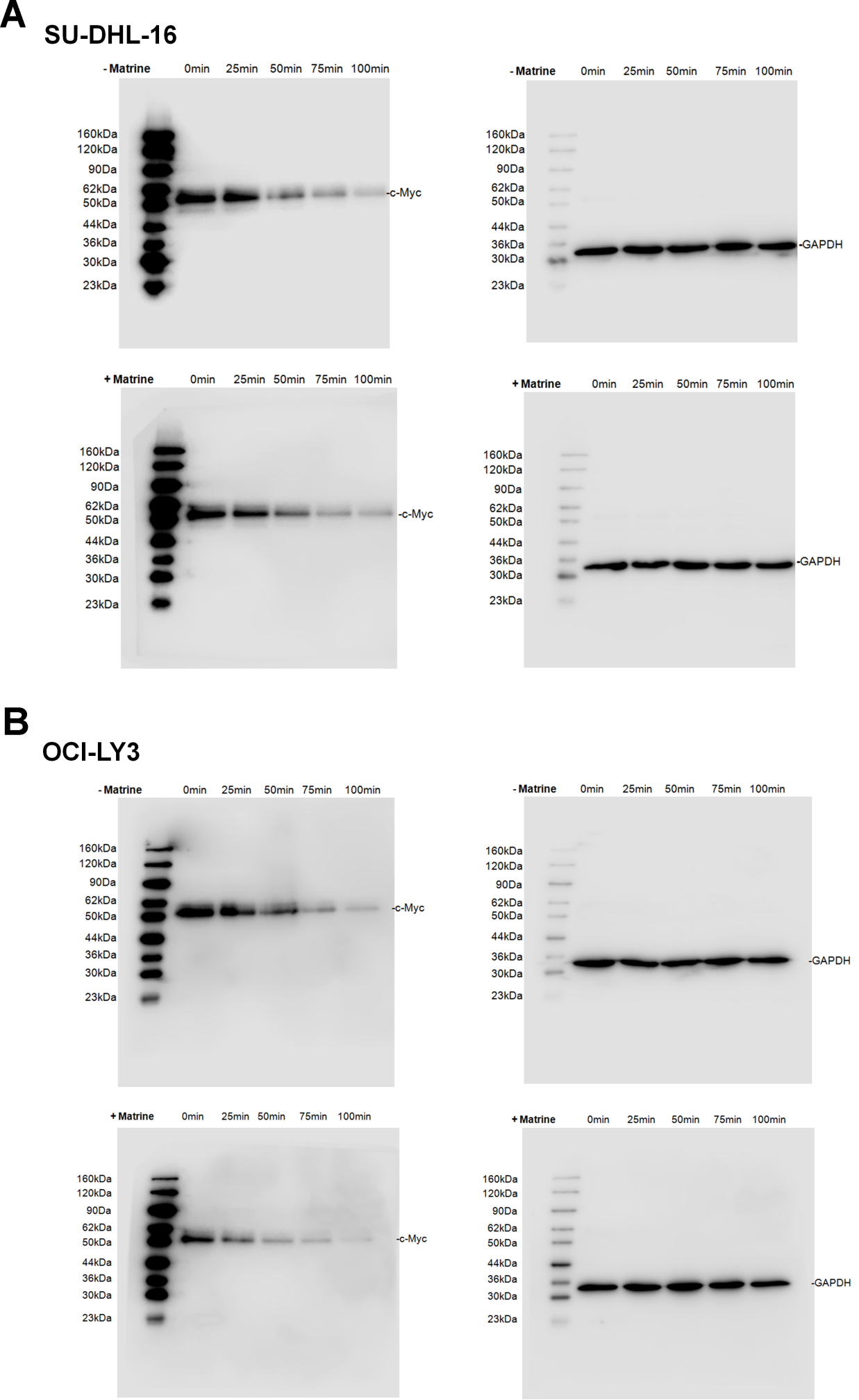


**Supplementary Figure 4**. Matrine promoted c-Myc protein degradation in DLBCL cells. Cycloheximide chase assay was used for the half-life of c-Myc protein. SU-DHL-16 and OCI-LY3 cells (1.25 × 10^6^) were treated with 1.76 mM and 4.1 mM matrine for 12 h, respectively. Cells were then treated with cycloheximide (100 μg/mL) for the indicated minutes, and western blotting was performed. Cells treated without matrine were used as control. (A) Representative WB results of c-Myc and GAPDH in the control and matrine-treated SU-DHL-16 cells, respectively. (B) Representative WB results of c-Myc and GAPDH in the control and matrine-treated OCI-LY3 cells, respectively.

**Supplementary Figure 5**


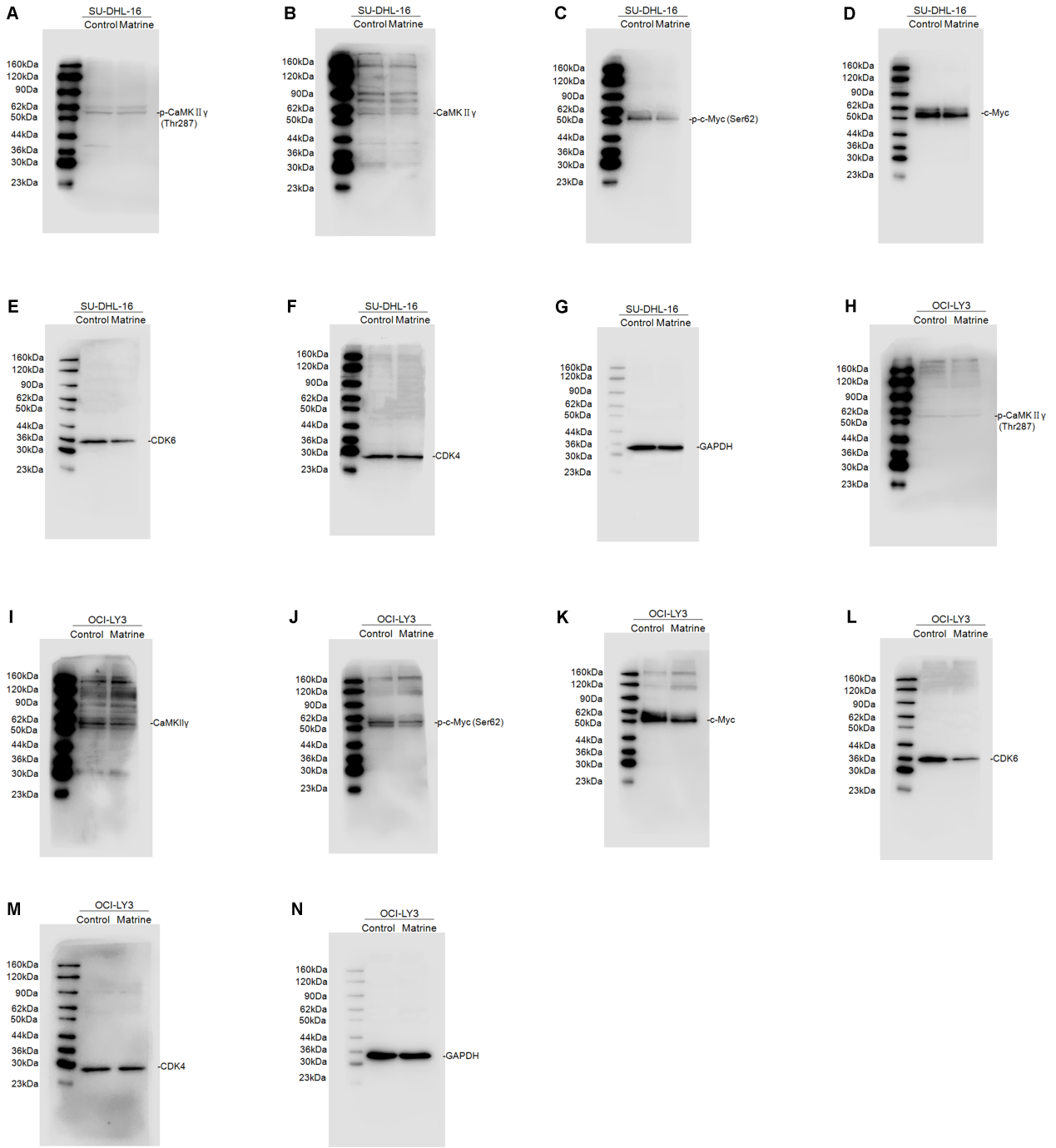


**Supplementary Figure 5**. Matrine inhibited the growth of DLBCL cells through CaMKIIγ/c-Myc/CDK pathway. SU-DHL-16 and OCI-LY3 cells (2.5×10^6^) were treated with matrine at 1.76 mM and 4.1 mM for 48 h, respectively, and followed by western blot. Cells treated without matrine were used as control. (A) Representative WB result of p-CaMKIIγ(Thr287) in SU-DHL-16 cells. (B) Representative WB result of CaMKIIγ in SU-DHL-16 cells. (C) Representative WB result of p-c-Myc (Ser62) in SU-DHL-16 cells. (D) Representative WB result of c-Myc in SU-DHL-16 cells. (E) Representative WB result of CDK6 in SU-DHL-16 cells. (F) Representative WB result of CDK4 in SU-DHL-16 cells. (G) WB result of GAPDH, the loading control for A, B, C, D, E and F. (H) Representative WB result of p-CaMKIIγ(Thr287) in OCI-LY3 cells. (I) Representative WB result of CaMKIIγ in OCI-LY3 cells. (J) Representative WB result of p-c-Myc (Ser62) in OCI-LY3 cells. (K) Representative WB result of c-Myc in OCI-LY3 cells. (L) Representative WB result of CDK6 in OCI-LY3 cells. (M) Representative WB result of CDK4 in OCI-LY3 cells. (N) WB result of GAPDH, the loading control for H, I, J, K, L and M.

**Supplementary Figure 6**


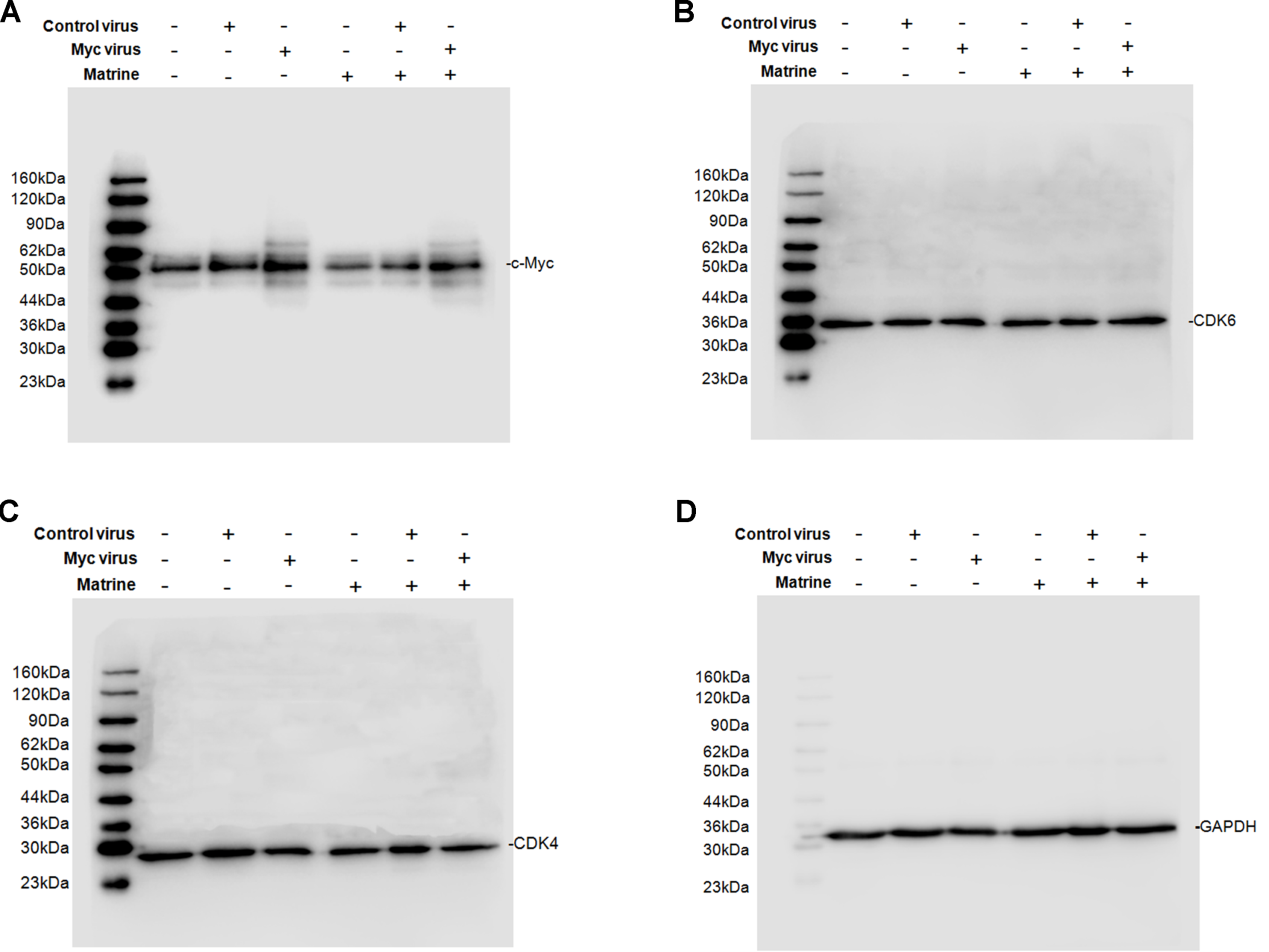


**Supplementary Figure 6**. Salvage of matrine-induced growth inhibition rescued by ectopic expression of c-Myc. SU-DHL-16 cells (1 × 10^6^) were treated with or without 1.76 mM matrine and recombinant c-Myc adenovirus or control adenovirus for 48 h, followed by western blot. Cells treated without matrine and adenovirus were used as control. (A) Representative WB result of c-Myc. (B) Representative WB result of CDK6. (C) Representative WB result of CDK4. (D) WB result of GAPDH, the loading control for A, B and C.
